# In-situ alignment of 3D printed anisotropic hard magnets

**DOI:** 10.1038/s41598-022-20669-8

**Published:** 2022-10-20

**Authors:** M. Suppan, C. Huber, K. Mathauer, C. Abert, F. Brucker, J. Gonzalez-Gutierrez, S. Schuschnigg, M. Groenefeld, I. Teliban, S. Kobe, B. Saje, D. Suess

**Affiliations:** 1 Faculty of Physics, University of Vienna, 1090 Vienna, Austria; 2 Platform MMM Mathematics–Magnetism–Materials, University of Vienna, 1090 Vienna, Austria; 3 Institute of Polymer Processing, Montanuniversitaet Leoben, 8700 Leoben, Austria; 4 Magnetfabrik Bonn GmbH, 53119 Bonn, Germany; 5 Department of Nanostructured Materials, Jožef Stefan Institute, 1000 Ljubljana, Slovenia; 6 Kolektor Magnet Technology GmbH, 45356 Essen, Germany; 7 Luxembourg Institute of Science and Technology, 4362 Esch-sur-Alzette, Luxembourg

**Keywords:** Engineering, Materials science

## Abstract

Within this work, we demonstrate in-situ alignment of the easy axis single-crystal magnetic particles inside a polymer matrix using fused filament fabrication. Two different magnetic materials are investigated: (i) Strontium hexaferrite inside a PA6 matrix, fill grade: 49 vol% and (ii) Samarium iron nitride inside a PA12 matrix, fill grade: 44 vol%. In the presence of the external alignment field, the strontium hexaferrite particles inside the PA6 matrix can be well aligned with a ratio of remnant magnetization to saturation magnetization in an easy axis of 0.7. No significant alignment for samarium iron nitride could be achieved. The results show the feasibility to fabricate magnets with arbitrary and locally defined easy axis using fused filament fabrication since the permanent magnets (or alternatively an electromagnet) can be mounted on a rotatable platform.

## Introduction

Additive manufacturing of magnetic materials including, soft magnetic materials, hard magnetic materials, shape memory alloys and graded magnetic alloys has recently received considerable attention^1^. A particular interesting possibility is the accelerated development of magnetic materials using high throughput methods based on additive manufacturing^2,3^. Another success story of additive manufacturing is the concept of mass customization^4^ which is used for example to realize plastic braces using 3D stereolithography^5^. The application of a magnetic customization concept was demonstrated by Huber et al.^6^ where the homogeneity of a magnetic field in a cavity (used for example to calibrate magnetic sensor) was improved using topology optimized and additively manufactured passive shimming elements.

In 2016 the fabrication of 3d printed permanent magnets has been reported for the first time. In the work of Huber et al. bonded Nd-Fe-B magnets have been realized using commercially available fused filament fabrication printers by using magnetic filaments composed of isotropic Nd-Fe-B particles embedded in a polymer matrix^7^. In the same year Paranthaman et al. realized bonded Nd-Fe-B magnets using binder jetting^8,9^. Nd-Fe-B bonded magnets with very high lateral resolution have been realized using stereolithography^10^. Dense Nd-Fe-B magnets without a polymer matrix have been fabricated using laser based additive manufacturing techniques^11,12^, where grain boundary diffusion process were used to enhance the coercivity of NdFeB magnets^13^.

In all these previous works on additive fabricated permanent magnets isotropic magnets were produced. In isotropic permanent magnets the remanence is significantly smaller than the saturation magnetization, which limits the performance of these magnets. A significant step forward is the fabrication of anisotropic magnets, where each magnetic particle is composed of a single magnetic crystal with a single easy axis. If an external field ***H***_ext_ is applied, a mechanical torque ***T*** acts on the particle. For a given external field ***H***_ext_ the mechanical torque can be calculated by first determining the micromagnetic equilibrium magnetization ***M***(***x***) within each particle. Then the mechanical torque ***T*** can be calculated by

1 $$ {\mathbf{T}} = \mu_{0} \int\limits_{{V_{particle} }} {{\mathbf{M}}\left( {\mathbf{x}} \right) \times {\mathbf{H}}_{ext} } dV, $$

where *V*_particle_ is the volume of the particle. The equilibrium direction of ***M***(***x***) depends on crystalline anisotropy, demagnetizing energy, which also gives rise to shape anisotropy, the exchange energy and of course on the external field ***H***_ext_. For single domain particles without shape anisotropy the mechanical torque tries to align the easy axis parallel to the external field ***H***_ext_. The counter acting torque is due to the viscosity and friction of molten compound. An alignment model that describes coupled particle–fluid-magnetic field interactions during additive manufacturing of anisotropic bonded magnets is presented by Sarker et al.^14^.

Experimentally alignment of fused filament was realized by Sonnleitner et al.^15^ by printing on the building platform where permanent magnets are located under the platform. Here the magnetic stray field of a permanent magnet aligns the particles during the printing process. An alignment after the fabrication process is reported by Gandha et al.^16^.

This paper presents a significant step forward by producing magnets where the easy axis can be locally aligned in-situ during the printing process. This is realized by redesigning the printer nozzle of a fused filament fabrication printer (FFF) to place permanent magnets next to the nozzle. In principle, this set-up of the permanent magnets can be realized on a rotational platform so that the magnetic field can be rotated during the printing process. Alternatively, electromagnets could be used to realize the required fields for the alignment^17^.

The magnetic stray field of the permanent magnets is optimized so that it aligns the easy axis of anisotropic ferromagnetic particles inside a paste-like compound material when it is in the molten state.

## Methods

The bonded magnets are printed with a commercially available Velleman’s K8200 printer. This printer works by use of the fusing filament fabrication (FFF) principle. The open source printer software Marlin is used to control the printer process. Printing temperature of up to 350 °C can be reached by using a E3D’s Titan Aero extruder.

The filaments consist of a compound of magnetic powders and the binder. Two different binders are used for the two different magnetic particles, in particular:

- Strontium-hexaferrite (SrFe_12_O_19_) inside a PA6 matrix (Sprox^®^ 10/20p), fill grade: 49 vol%
- Samarium-iron-nitride (Sm_2_Fe_17_N_3_) inside a PA12 matrix, fill grade: 44 vol%.

The Sprox^®^ compound is prefabricated by Magnetfabrik Bonn. This strontium hexaferrite powder consists of cuboid flakes with approximate dimensions in the range 6 × 2 × 2 µm^3^ with *B*_r_ = 196 mT, and with *H*_cJ_ = 183  kA/m. The Sprox^®^ compound is prefabricated by Magnetfabrik Bonn. The ${\mathrm{Sm}}_{2}{\mathrm{Fe}}_{17}{\mathrm{N}}_{3}$ particles are spherical with a diameter in the range of 2–4 μm with *B*_r_ = 1.31 T and *H*_cJ_ = 889  kA/m The magnetic filaments were produced at the Institute for Polymer Processing by a screw extruder^18^.

Further details on the used filaments including scanning electron microscopy images of the filaments can be found in Ref.^15^.

**Figure 1:**
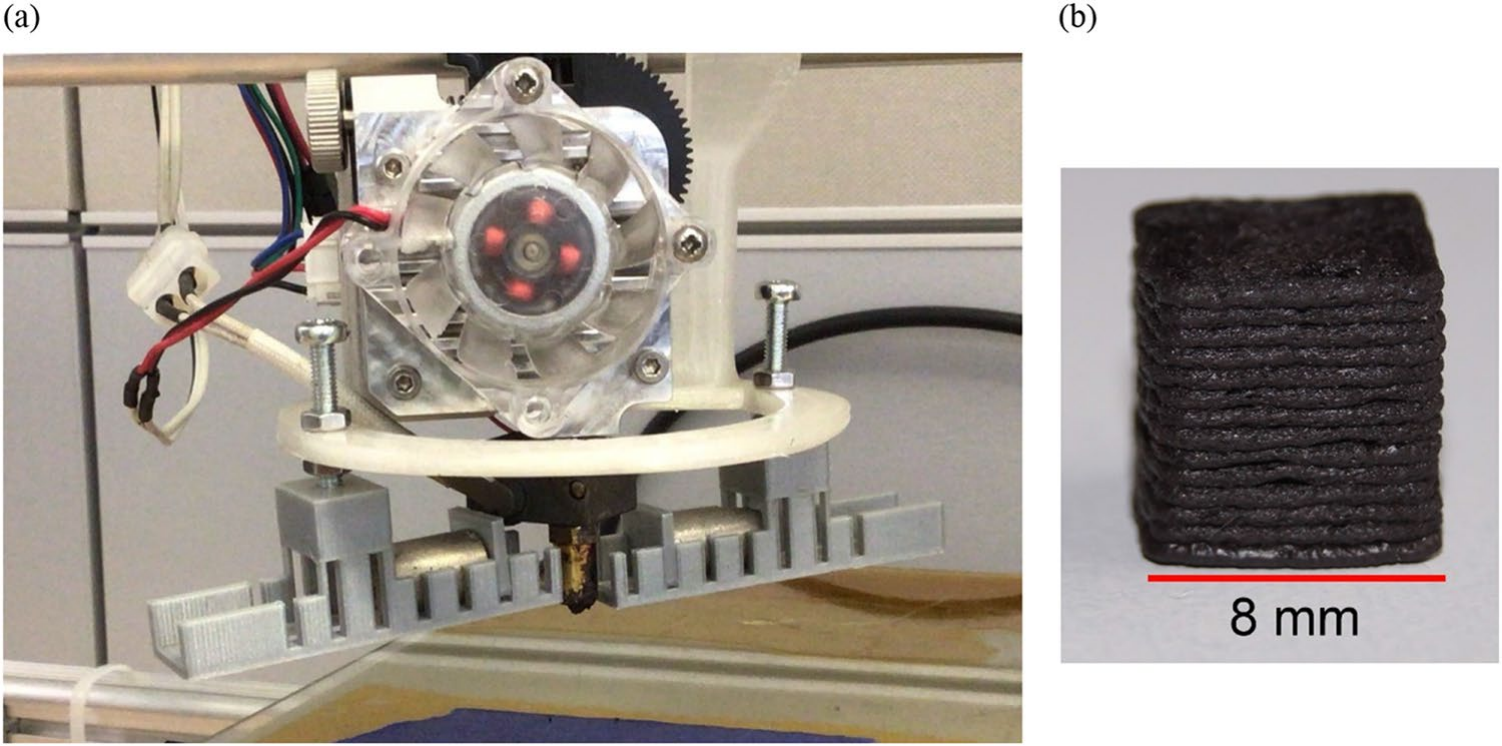
(**a**) Modified 3D-Printer showing the extruder including the nozzle and the flexible fixing unit for the permanent magnets (**b**) printed SrF_12_O_19_ cube (8 mm × 8 mm × 8 mm).

**Figure 2:**
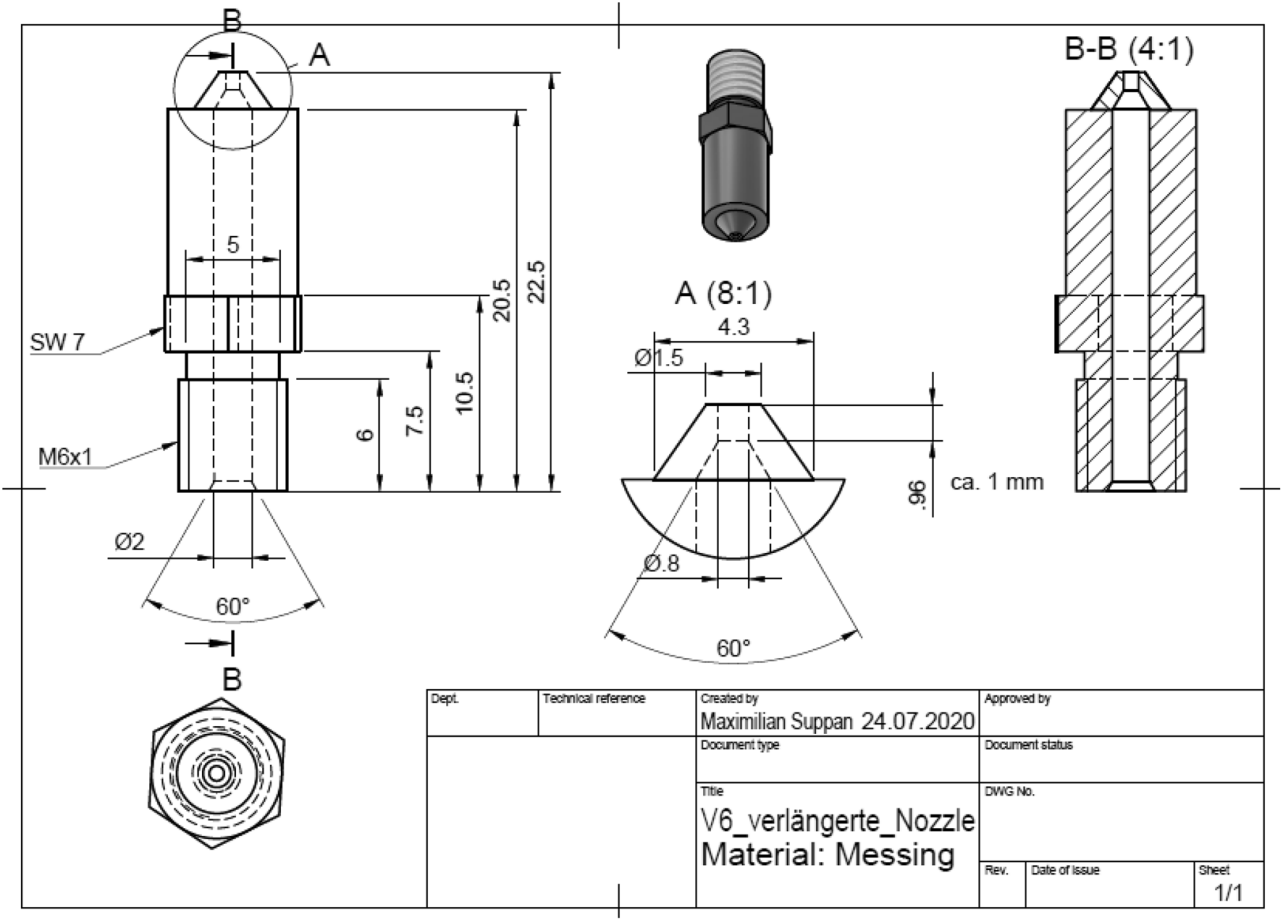
Detailed design of the developed magnetic nozzle optimized for the alignment process. Dimensions are in mm. The material is brass.

This printer was extended with an alignment unit containing permanent magnets as shown in Fig. 1a. The printing nozzle is specially designed to optimize the alignment process as shown in Fig. 2.

The magnetic characterization was done using vibrating sample magnetometer (VSM—Quantum Design PPMS-9 Tesla). The large bore option of the quantum PPMS is used. The measurements are done at room temperature.

## Results

The ${\mathrm{Sm}}_{2}{\mathrm{Fe}}_{17}{\mathrm{N}}_{3}$ particles are spherical with a diameter in the range of 2–4 μm The magnetic filaments were produced at the Institute for Polymer Processing by a screw extruder^18^.The Sprox^®^ compound is prefabricated by Magnetfabrik Bonn. This strontium hexaferrite powder consists of cuboid flakes with approximate dimensions in the range of 6 × 2 × 2 µm^3^ with *B*_r_ = 196 mT, and with *H*_cJ_ = 183 kA/m. In order to realize the alignment of the magnetic particles, two cylindrical Sm_2_Co_17_ permanent magnets with a diameter of 12 mm and a height of 18 mm are placed next to the nozzle. The magnetic field generated from the magnets aligns the magnetic particles of the melted filament within the nozzle when the polymer is in the liquid state. The magnetic properties of the used Sm_2_Co_17_ permanent magnets are summarized in Table 1.

**Table 1:** Magnetic properties of the Sm_2_Co_17_ permanent magnets that are placed next to the nozzle in order to align the magnetic particles in-situ.

${B}_{r}$	$1 100 \; \mathrm{mT}$
${H}_{cJ}$	$1194 \; \mathrm{kA}/\mathrm{m}$
$\rho$	$8.4 \; \mathrm{g}/{\mathrm{cm}}^{3}$
${\left(BH\right)}_{max}$	$240 \; \mathrm{kJ}/{\mathrm{m}}^{3}$
${T}_{C}$	$820\;^\circ \mathrm{C}$

**Figure 3:**
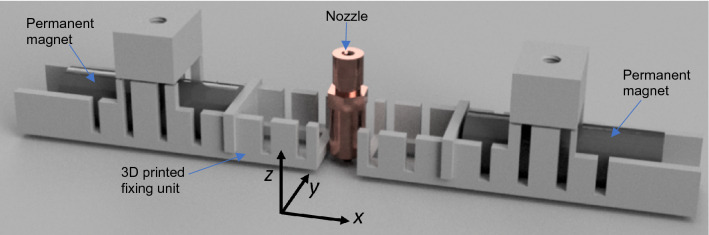
Flexible 3d printed fixing unit for the bias magnets that is placed next to the nozzle.

Detailed optimization of the field strength during the printing of the magnet is essential. Too small fields cannot align the particles, too large fields attract the magnetic particles towards the poles of the permanent magnets, and no printed object can be realized. To be able to adjust the field, a flexible fixing unit for the magnets was printed from polylactides, as shown in Fig. 3. Depending on the required field strength, the distance between the two cylindrical magnets was adjusted. In Fig. 1a, the modification of the 3D-Printer is illustrated. The printer has a spacious scaffold out of aluminum, which provides sufficient space to attach the fixing units around the printhead.

The field can be varied by manually changing the distance of the permanent magnets. Due to the attraction of the printing material by the magnets, the magnets could not be placed at the minimum distance with no gap between the nozzle and the magnets. The distance has to be increased until the material remains on the printing bed during the printing process. The impact of the field was significantly different for the two investigated materials. The ${\mathrm{Sm}}_{2}{\mathrm{Fe}}_{17}{\mathrm{N}}_{3}$ filament requires a larger distance between the magnets to avoid that the printed filament being attracted to the poles of the permanent magnets, which is due to the higher saturation magnetization compared to SrFe_12_O_19_. The magnetic field strength in the printing nozzle has been found by simulations with COMSOL Multiphysics. For the simulation parameters, the magnetic properties as shown in Table 1 are used where for the SrFe_12_O_19_,a distance of 15 mm is used and for the ${\mathrm{Sm}}_{2}{\mathrm{Fe}}_{17}{\mathrm{N}}_{3}$,a distance of 21 mm is used (Table 2).

**Table 2:** Printing parameters for the two used materials.

Parameter	SrFe_12_O_19_ + PA6	${\mathrm{Sm}}_{2}{\mathrm{Fe}}_{17}{\mathrm{N}}_{3}+\mathrm{PA}12$
Distance	15 mm	21 mm
*B*_x_	150–160 mT	90–100 mT
Printing temperature	295 °C	260 °C
Print bed temperature	40 °C	60 °C
Nozzle diameter	0.8 mm	0.8 mm
Print speed	25 mm/s	25 mm/s
Filling	100% rectilinear	100% rectilinear
Layer height	0.1 mm	0.15 mm
Filament diameter	1.75 mm	1.75 mm

**Figure 4:**
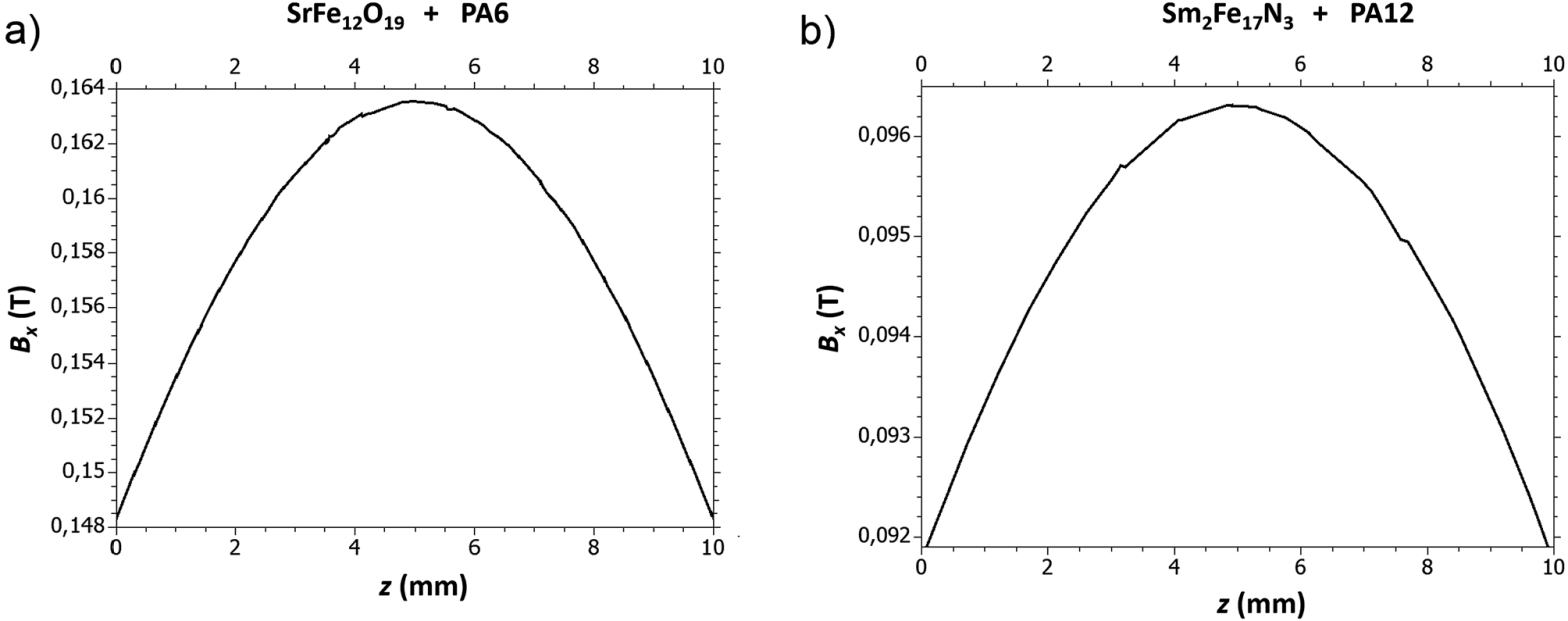
Magnetic flux density B_x_ as function of distance to the building platform (z-direction is equal to the building platform normal). The zero of z = 0 is the end of the nozzle which is equal to the position of the building platform. (**a**) The distance between the magnets is 15 mm (suitable for Sprox 10/20p); (**b**) the disctance between the magnets is 21 mm (suitable for ${\text{Sm}}_{2}{\text{Fe}}_{17}{\text{N}}_{3}$ ).

In Fig. 4, the strength of the B_x_ field is shown as a function of the z distance. The z coordinate is zero at the end of the nozzle, which is equal to the position of the building platform.

The settings for the manufacturing process with the 3D-printer were found empirical. In Table 2, these parameters are listed.

The fabricated samples are cubes with an edge length of 8 mm In Fig. 1b a printed SrFe_12_O_19_ + PA6 cube is shown. The rectilinear filling is rotated by 90° by adjacent layers so that no flow anisotropy occurs. The magnetic characteristics were measured with a vibrating sample magnetometer. For this purpose, the samples were cut into smaller cubes to be sufficient small for the VSM. In order to evaluate the degree of the alignment, the ratio of remanence to saturation magnetization *M*_s_ is used. The Stoner-Wohlfarth model of non-interacting particles predicts that an object with fully parallel aligned domains has a remanent magnetization ${M}_{r}$ that is twice as big as ${M}_{r}$ of an assembly of randomly oriented domains^19^. Hence, the quotient *M*_r_/*M*_s_ shows the degree of the alignment of the particles. If it is fully aligned, the quotient *M*_r_/*M*_s_ = 1. Of course, a high degree of alignment is beneficial since it leads to higher stray fields for the applications.

**Figure 5:**
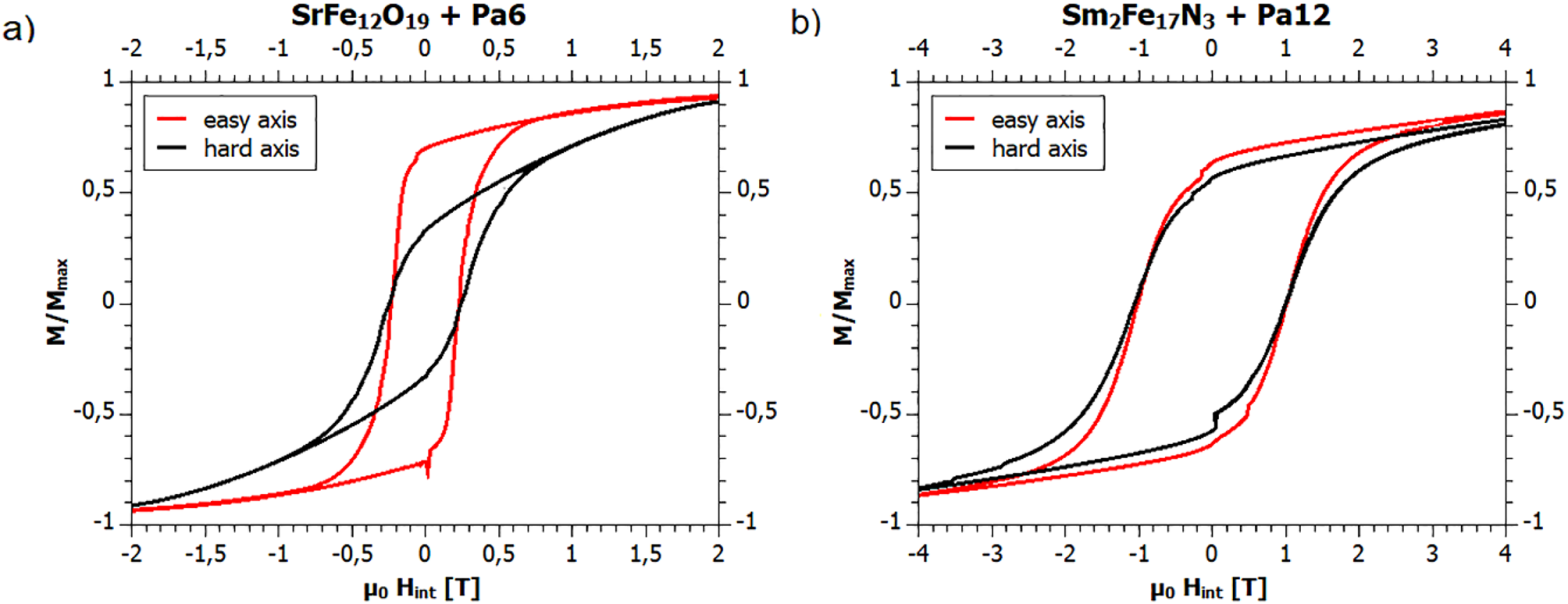
Hard axis and easy axis hysteresis loops of the (**a**) SrFe_12_O_19_ (Strontium hexaferrite) and (**b**) Sm_2_Fe_14_N_3_. Here the easy axis loop denotes the direction that is parallel to the applied field during the printing process.

In Fig. 5a, the normalized magnetization *M*_r_/*M*_s_ of the SrFe_12_O_19_ sample depending on the internal magnetic field ${{\mu }_{0}H}_{int}$ is shown. ${H}_{int}$ is the magnetic field after the magnetization correction ${H}_{int }={H}_{d}+{H}_{ext}$ with the demagnetization field ${H}_{d}=-{N}_{d}M$. The demagnetizing factor depends on the shape of the sample and is about *N*_d_ = 1/3 for a cube. In Fig. 5b, the same loops for the ${\mathrm{Sm}}_{2}{\mathrm{Fe}}_{17}{\mathrm{N}}_{3}+\text{PA}12$ sample are shown.

For SrFe_12_O_19_, the ratio *M*_r_/*M*_s_ in the direction of the easy axis is $0.70,$ and along the hard axis, it is 0.33. Here, we denote the direction in which the field was applied during printing as the easy axis. For ${\mathrm{Sm}}_{2}{\mathrm{Fe}}_{17}{\mathrm{N}}_{3}+\text{PA}12$ the normalized magnetization along the easy axis is 0.65 and 0.55 in the direction of the hard axis.

The results of the two materials are remarkable different, which was about to expect concerning the intrinsic magnetic properties of both materials. For the ferrite material, the particles could be significantly aligned. For the rare earth bonded magnet the measurements along the easy and hard axis are very similar, which implies that the field’s strength was too small to align the powder along their easy axis. For the aligning of this material, a field of about 1 T, or 15 times what was possible, would be necessary according to Ref.^16^. However, for large fields, we obtain the problem that the melted filament is attracted to the poles of the permanent magnet instead of the building platform. In future work one might work on more homogenous fields, so that the field gradients are smaller and the attractive force on the melted filament becomes smaller. Such a field might be realized by larger field sources such as larger permanent magnets.

## Conclusion

In conclusion, this paper presents a newly designed 3D-Printer, which allows us to produce anisotropic polymer-bonded magnets by aligning ferrite powder during the printing process. It is possible to print complex structures with special magnetic capabilities. It is easy to modify the construction of the fixing units of the permanent magnets so that they can be rotated and any direction of the alignment within the plane can be achieved. Hence, highly optimized magnets can be produced, such as Halbach arrays which cannot be produced by any other method.

## Data Availability

The datasets used and/or analysed during the current study available from the corresponding author on reasonable request.
